# Modulating Near-Infrared Persistent Luminescence via Diverse Preparation Approaches

**DOI:** 10.3390/nano14191613

**Published:** 2024-10-09

**Authors:** Xiaomeng Wang, Hengli Zhu, Yan Liu, Jingyuan Li, Lejia Cao, Jiaren Du, Hengwei Lin

**Affiliations:** International Joint Research Center for Photo-Responsive Molecules and Materials, School of Chemical and Material Engineering, Jiangnan University, Wuxi 214122, China; 7220611009@stu.jiangnan.edu.cn (X.W.); 6230609013@stu.jiangnan.edu.cn (H.Z.); 1052220206@stu.jiangnan.edu.cn (Y.L.); 1052220130@stu.jiangnan.edu.cn (J.L.); 1052210127@stu.jiangnan.edu.cn (L.C.)

**Keywords:** persistent luminescence, near-infrared emission, ZnGa_2_O_4_:Cr^3+^, trap distribution manipulation

## Abstract

Near-infrared (NIR) persistent luminescence (PersL) materials have attracted extensive attention due to their great promise in medical diagnostics, bio-imaging, night vision surveillance, multi-level anticounterfeiting, and information encryption. To achieve NIR PersL (micro/nano-) materials with the desired properties, a variety of synthesis methods have been employed, including solid-phase reaction and liquid-phase synthesis. Different synthesis methods have different but important effects on the micro/nano-structure, luminescence, and PersL properties of the materials. Moreover, the influence of various synthesis methods on the properties of NIR PersL materials determines the selection of preparation approaches for other new material systems. Taking the representative NIR PersL ZnGa_2_O_4_:Cr^3+^ material as an example, four synthesis procedures are applied, namely, high-temperature solid-state reaction (SSR), high-temperature molten salt method (MSM), hydrothermal method (HM), and microwave-assisted solid-state (MASS) method. The structural and luminescent properties of samples made by SSR, MSM, HM, and MASS are compared. Notably, it is revealed that the MASS method can create additional trapping energy levels, which is of great significance for emerging applications. This work demonstrates the different effects of synthesis methods on PersL performance and provides a good guideline for the rapid and reasonable selection of preparation methods for diverse applications.

## 1. Introduction

Persistent luminescence (PersL) is a distinct optical phenomenon in which light emission continues for several seconds, minutes, or hours after the stoppage of excitation sources [1,2,3,4]. PersL materials have been widely applied in security signage and night vision due to the time-delayed light emission [5,6]. Examples are CaAl_2_O_4_: Eu^2+^, Nd^3+^, SrAl_2_O_4_:Eu^2+^, Dy^3+^ (SAO: Eu^2+^, Dy^3+^), Sr_4_Al_14_O_25_:Eu^2+^, Dy^3+^ (SAO25: Eu^2+^, Dy^3+^), Y_2_O_2_S: Eu^3+^, Mg^2+^, Ti^4+^, and ZnGa_2_O_4_:Cr^3+^ (ZGO: Cr^3+^), which enable a variety of functionality in low-intensity light conditions [7,8,9,10]. In recent years, near-infrared (NIR) technology has emerged as a crucial facilitator in the areas of food quality analysis, night vision, information encryption, and biological imaging, benefiting from its advantages of high penetration, invisibility to the naked eye, and low absorption by biological tissue [11,12,13,14,15]. Due to the time-delayed light emission, NIR PersL materials with a high signal-to-noise ratio show great potential in NIR technologies; therefore, rational design and facile preparation of high-performance NIR PersL materials are crucial for emerging applications [16,17].

The synthesis method for PersL materials plays a significant role in micro/nano-structure, luminescence, and PersL properties. The conventional synthesis method is the high-temperature solid-state reaction (SSR), which usually requires a long reaction time and a high sintering temperature. This SSR method is widely used due to its straightforward synthesis procedure and simple operation [18]. To prevent the non-oxide materials from being oxidized during the SSR procedure, Dash et al. presented a molten salt method (MSM) to synthesize the oxidation-prone materials, which employed molten salts, as a reaction medium, to protect the non-oxide powders from oxidation in air during the high-temperature process. The total synthesis time was greatly shortened and the temperature required for a completed reaction was lowered [19]. Recently, Peng and co-workers have applied MSM to make mechanoluminescence (ML) materials. The biphase ZnS and CaZnSO systems were obtained by MSM with excellent ML properties [20]. Furthermore, the ZnGa_2_S_4_: Mn phosphors with deep-red ML were obtained using the MSM method, and the relationship between ML performance and crystal structure was elucidated [21]. These results demonstrate the potential of the MSM method in the synthesis of luminescent materials. In addition to the MSM, the microwave-assisted solid-state method (MASS) has been an efficient and facile synthesis method for inorganic luminescent materials in recent years. Miranda de Carvalho et al. first synthesized a series of the photochromic Na_8_Al_6_Si_6_O_24_ (Cl, S)_2_ compounds using the MASS method. It was proven that the structural conversion from Zeolite A to hackmanite minerals through an aluminosilicate crystalline intermediate with the aid of MASS preparation is very effective [22]. Subsequently, Poelman and co-workers employed the MASS approach to successfully prepare aluminates, germanates, and gallates. These works show the feasibility and advantages of MASS in improving PersL performance [23,24,25]. Apart from the above-mentioned solid-phase preparations, the hydrothermal method (HM) in liquid-phase reaction is also a common synthesis process for preparing luminescent (nano-) materials. Li et al. first reported a direct aqueous-phase chemical synthesis route for NIR PersL nanoparticles. They obtained the ZGO: Cr^3+^ nanoparticles with sub-10 nm sizes by adjusting the molar ratio of Zn/Ga in a direct hydrothermal process [26]. Thereafter, Arroyo et al. used ZGO: Cr^3+^ particles prepared by HM to obtain transparent films on a quartz substrate, which shows the great application potential in chemical sensing, information storage, labeling, and anti-counterfeiting technology [27].

Although there are many methods for synthesizing PersL materials, one application purpose is inclined to a certain method for a desirable morphology or optical performance. For example, in vivo imaging applications require nanosized products from a synthesis method because using very large particles for intravenous injections of organisms could block blood vessels and cause serious rejection by the body. For the purpose of information encryption, a large number of widely distributed traps can be obtained from multi-mode luminescence materials, so that multi-dimensional information encryption can be realized. Therefore, the study of the effects of different synthesis methods on NIR PersL properties is beneficial to the rapid development of other material systems and reasonable selection for diverse applications.

**Figure 1:**
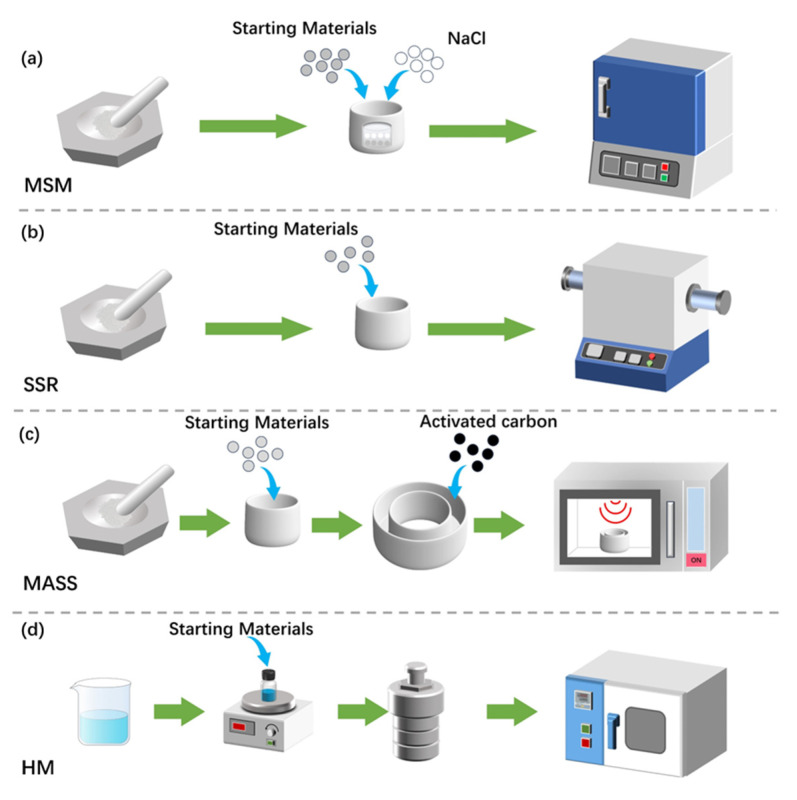
Diagrammatic sketch of various synthesis approaches for preparing ZGO: Cr^3+^. (**a**) Molten salt method (MSM), (**b**) Solid-state reaction (SSR), (**c**) Microwave-assisted solid-state (MASS), and (**d**) Hydrothermal method (HM).

In this work, the widely studied ZGO: Cr^3+^ NIR PersL material is selected as the research object. Different synthesis methods may affect material properties under varying conditions. Four representative methods were employed. In addition to the common SSR and HM preparation methods in ZGO, MSM, which has the advantages of the solid–liquid phase method, and the emerging MASS method, have been introduced. The detailed synthesis process for obtaining ZGO: Cr^3+^ is shown in Figure 1 and detailed information on the synthesis procedure can be found in the experimental section. The impact of the synthesis methods on the optical properties of ZGO: Cr^3+^ was explored by focusing on its micro/nano-structure, optical properties, and the corresponding trap distribution. It is found that the MASS method can create additional trapping levels. Based on the performance differences of ZGO: Cr^3+^ obtained by different methods, the possibility of different methods preferentially applied in different fields in material development is discussed. This may open a frontier in the development of new materials and emerging applications.

## 2. Materials and Methods

### 2.1. Materials Preparation

#### 2.1.1. The MSM Samples

As Figure 1a shows, for the MSM process, the starting materials, ZnO (99.9%, Macklin, Shanghai, China), Ga_2_O_3_ (99.99%, Aladdin, Shanghai, China), and Cr_2_O_3_ (99.95%, Aladdin, Shanghai, China), were weighed according to stoichiometric ratios. The amount of Cr^3+^ dopant was kept at 1% mole with respect to the content of Ga in the ZGO matrix for all the samples. The NaCl (99.99%, Aladdin, Shanghai, China) was added to the raw materials and the mixture was ground in an agate mortar. Subsequently, the aluminum crucibles were used to hold the mixture. The mixture was sintered in air at 1000 °C in a muffle furnace for 2 h at a heating rate of 5 °C min^−1^. The sample was then cooled down to room temperature in the furnace, and the obtained powder was fully ground again. The powder was centrifuged at 8000 rpm several times in distilled water to remove sodium salts and residuals, and then thoroughly washed for further analysis.

#### 2.1.2. The SSR Samples

The SSR procedure is shown in Figure 1b. The starting materials, ZnO (99.9%, Macklin, Shanghai, China), Ga_2_O_3_ (99.99%, Aladdin, Shanghai, China), and Cr_2_O_3_ (99.95%, Aladdin, Shanghai, China), were weighed according to stoichiometric ratios. The precursors were sintered in air at 1300 °C in a muffle furnace for 6 h at a heating rate of 5 °C min^−1^. The sample was then cooled down to room temperature in the furnace, and the obtained powder was fully ground for further analysis.

#### 2.1.3. The MASS Samples

The MASS procedure is shown in Figure 1c. The starting materials, ZnO (99.9%, Macklin, Shanghai, China), Ga_2_O_3_ (99.99%, Aladdin, Shanghai, China), and Cr_2_O_3_ (99.95%, Aladdin, Shanghai, China), were weighed according to stoichiometric ratios. The home-built microwave reaction device was composed of two alumina crucibles and aluminum silicate insulation bricks. The larger crucible (50 mL) holds the activated carbon (200 mush, Leyan, Shanghai, China), while the smaller crucible (5 mL) contains 0.8 g of the precursor. The smaller crucible was covered with an alumina tray and inserted into the activated carbon within the larger crucible. The inner and smaller crucible was tightly covered with an alumina disk to hold the reaction temperature and prevent contact with the volatilized carbon. There were 14 g of activated carbon in the larger crucible. The two crucibles were then placed into the cavity of a high-temperature aluminosilicate insulation brick. The materials were irradiated in a lab microwave oven (frequency 2.45 GHz) for 25 min with a microwave power of 700 W. The obtained powder was fully ground for further analysis.

#### 2.1.4. The HM Samples

The HM process is illustrated in Figure 1d. A total of 0.2974 g of Zn(NO_3_)_2_·6H_2_O (99%, Aladdin, Shanghai, China), 0.5115 g of Ga(NO_3_)_3_·9H_2_O (99.9%, Aladdin, Shanghai, China), and 0.0041 g of Cr(NO_3_)_3_·9H_2_O (99%, Innochem, Beijing, China) were dissolved in 15 mL distilled water to form a mixture. The ammonia liquor was added to the above-mentioned mixture to adjust the pH to 9–9.5. The mixture was stirred intensely and then transferred into the Teflon-lined autoclave (25 mL) and tightly sealed. After a 10 h reaction at 180 °C in an oven, the autoclave was naturally cooled down to room temperature. Finally, the sample was deposited and washed with excess isopropanol. The white crystalline powders of ZGO: Cr^3+^ were obtained via drying at 50 °C in an oven.

#### 2.1.5. SAO: Eu^2+^, Dy^3+^ and SAO25: Eu^2+^, Dy^3+^ Phosphors

The SAO: Eu^2+^, Dy^3+^, and SAO25: Eu^2+^, Dy^3+^ phosphors were prepared by the MASS method mentioned above. SrCO_3_ (99.9%, Aladdin, Shanghai, China), Al_2_O_3_ (99.9%, Aladdin, Shanghai, China), Eu_2_O_3_ (99.99%, Aladdin, Shanghai, China), and Dy_2_O_3_ (99.99%, Aladdin, Shanghai, China), used as starting materials, were weighed. SAO: Eu^2+^, Dy^3+^: SrCO_3_ (1.4762 g), Al_2_O_3_ (1.0195 g), Eu_2_O_3_ (0.0087 g), Dy_2_O_3_ (0.0093 g). SAO25:Eu^2+^, Dy^3+^: SrCO_3_ (2.9525 g), Al_2_O_3_ (3.5686 g), Eu_2_O_3_ (0.0087 g), Dy_2_O_3_ (0.0093 g). The MASS procedure was similar to the preparation step for the MASS ZGO: Cr^3+^ samples. Microwave irradiation was performed for 25 min in a lab microwave oven (frequency 2.45 GHz) to prepare SAO: Eu^2+^, Dy^3+^, and SAO25: Eu^2+^, Dy^3+^ phosphors. The obtained powders were fully ground for further use.

#### 2.1.6. The Ball Milled ZGO: Cr^3+^ Phosphors

The ZGO: Cr^3+^ phosphors were further ball milled in a planetary ball milling equipment (MITR, Changsha, China). The ball milling process was performed using hard zirconia balls for thorough milling in an ethanol medium. A cycle of the milling process included a 30 min rotation at a speed of 300 rpm, followed by a 30 min suspension period. The cycle was repeated 2, 8, 20, and 34 times throughout the whole ball milling process.

### 2.2. Structural and Morphological Characterization

The crystallographic phase purity of the obtained phosphors was examined by X-ray diffraction (XRD) using an X-ray powder diffractometer (D8, Bruker AXS GmbH, Bruker, Germany) with Cu Kα1 (1.5406 Å) radiation (40 kV, 40 mA) at room temperature. The morphology of samples prepared using the four different synthesis methods was characterized using field emission scanning electron microscope (FESEM) and transmission electron microscopy (TEM). The morphologies of samples synthesized using the MSM, SSR, and MASS methods were analyzed using FESEM (S4800, Hitachi, Tokyo, Japan), while samples synthesized using the HM method were analyzed using TEM (JEM-2100PLUS, JEOL, Tokyo, Japan). The elemental analysis of ZGO: Cr^3+^ prepared by MASS and MSM was characterized using an energy dispersive spectrometer (EDS) equipped with FESEM (Regulus 8100, Hitachi, Tokyo, Japan).

### 2.3. Luminescence Characterization

The room temperature steady-state photoluminescence (PL), photoluminescence excitation (PLE) spectra, and luminescence decay profiles were measured by a fluorescence spectrometer (FS5, Edinburgh, UK) with a monochromated 450 W Xenon arc lamp as the excitation source. The diffuse reflection spectra (DRS) were obtained by a UV–vis spectrophotometer (UV-2700, Shimadzu, Kyoto, Japan). Thermoluminescence (TL) was measured by an SL08 TL spectrometer (Rongfan Tech., Guangzhou, China) equipped with R928 photomultiplier tubes (Hamamatsu Photonics, Japan). During the TL measurement, the 254 nm light-emitting diode (LED) was used as the excitation source, and the heating rate was kept at *β* of 1 °C s^−1^. The relevant traps were first emptied before each TL test. Photographs of the powders under daylight, 365 nm, and 254 nm were taken with a mirrorless camera (GX9, Lumix, Japan) in raw mode.

### 2.4. Night-Vision Surveillance Application

A series of ZGO: Cr^3+^ phosphors were ground to obtain the fine and homogenous powders. The patterns of Wafer, Leaf, Cloud, and Snowflake were made by plate metal combining SAO: Eu^2+^, Dy^3+^, SAO25: Eu^2+^, Dy^3+^, and ZGO: Cr^3+^ PersL phosphors. The emission images of all patterns were taken using Vision Camera (EOS Rebel T5i, Canon, Japan) and NIR Camera (LD-SW640171550, Xi’an Leading Optoelectronic Tech. Co., Ltd., Xi’an, China). The 365 nm LED was used as the excitation source. The afterglow (AG) images were also taken using Vision Camera and NIR Camera. The photographs of the patterns were taken after a 2 min delay time after the UV irradiation.

## 3. Results and Discussion

**Figure 2:**
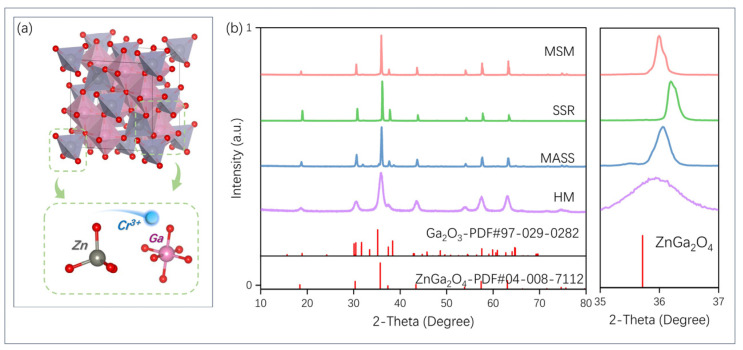
(**a**) Crystal structure of ZnGa_2_O_4_ with [ZnO_4_] and [GaO_6_] units; (**b**) XRD patterns of ZnGa_2_O_4_ prepared by MSM, SSR, MASS, and HM. The corresponding standard PDF card data for ZnGa_2_O_4_ (PDF#04-008-7112) and Ga_2_O_3_ (PDF# 97-029-0282) are given as a reference.

The crystal structure of ZnGa_2_O_4_ and the local coordination of Zn and Ga sites with four and six oxygen atoms are illustrated in Figure 2a. ZnGa_2_O_4_ possesses a spinel structure and belongs to the Fd3m (227) space group. Zn^2+^ ions adopt a 4-coordination, forming a tetrahedral structure denoted as [ZnO_4_], while Ga ions prefer a 6-coordination to form an octahedral structure. Due to the similar ionic radii between Cr^3+^ and Ga^3+^ ions in octahedral coordination, it is generally believed that Cr^3+^ tends to occupy the sites of Ga^3+^, serving as the luminescence center. XRD was conducted to investigate the influence of various synthesis methods on the crystal structure of ZGO: Cr^3+^. As shown in Figure 2b, the samples synthesized via MSM, SSR, MASS, and HM were compared with the standard patterns of ZnGa_2_O_4_ (PDF#04-008-7112). The results indicated that all samples were pure except for the MASS samples, which exhibited incomplete Ga_2_O_3_ at 32° and 38°. The effects of different synthesis methods on lattice constants were further investigated. The diffraction peaks of all samples shifted to a larger angle near 36°, possibly due to slight cell shrinkage caused by Cr^3+^ doping. The lattice constants varied depending on the synthesis method. In the MSM, SSR, MASS, and HM samples, the (311) crystal planes are located at 35.9°, 36.2°, 36.1°, and 35.8°, respectively, suggesting that the SSR synthesis process may cause noticeable shrinkage of the crystal cell volume. The effect of HM on cell volume is not significant. The corresponding crystallinity could be calculated by the following equation:

*Xc* = *Ic*/(*Ic* + *Ia*) (1)

where *Xc* represents crystallinity, *Ic* represents the diffraction integral intensity of crystalline, and *Ia* represents the diffraction integral intensity of amorphous. The crystallinity of samples synthesized by MSM, SSR, MASS, and HM was calculated as 93%, 95%, 97%, and 82%. It is noteworthy that the sample synthesized by HM exhibits the lowest crystallinity among the four methods. The small particle size as well as the low crystallinity obtained by HM is attributed to the low concentrations of the reagents, the relatively low temperature, the low solubility of the substance, the large volume of the dispersion medium separating the individual particles, the participation of water in the crystallization reaction, and other factors [28,29,30]. Subsequent TEM images further confirm the presence of nanoparticles obtained by HM.

**Figure 3:**
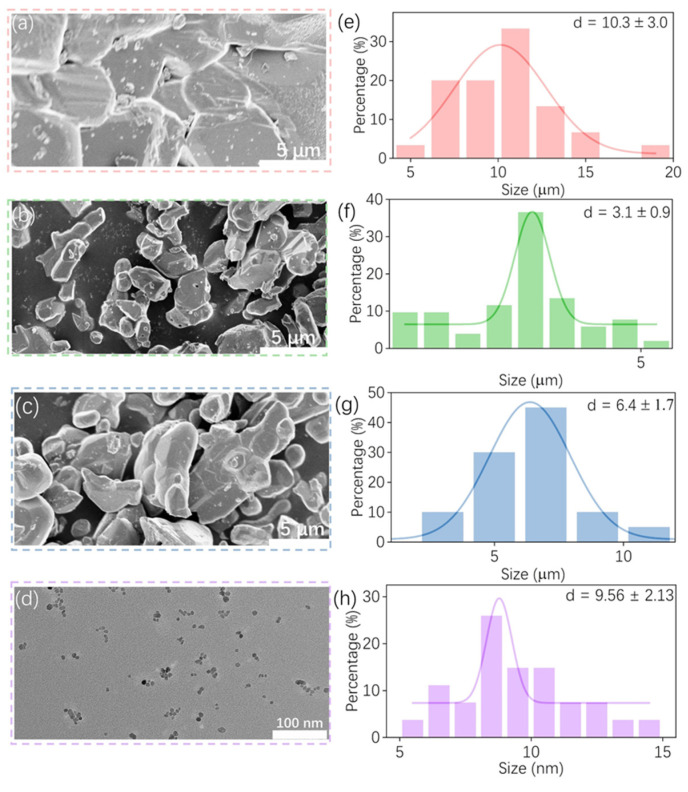
FESEM images of ZGO: Cr^3+^ synthesized by (**a**) MSM, (**b**) SSR, and (**c**) MASS, and TEM images of (**d**) HM. The corresponding statistics of particle sizes with fitting plots are shown in (**e**), (**f**), (**g**), and (**h**), respectively.

To further investigate the impact of various synthesis methods on crystal micro/nano-structure, FESEM and TEM images with corresponding grain sizes are presented in Figure 3. The FESEM and TEM images of different magnifications are also shown in Figure S1. The typical FESEM images of samples synthesized by MSM, SSR, and MASS are depicted in Figure 3a–c, respectively. Combining the FESEM image shown in Figure S1a–c, the microstructures of these samples exhibit random particle distributions, with average sizes recorded in Figure 3e,f at approximately 10.3, 3.1, and 6.4 μm, respectively. The grain size of the MSM sample is notably larger than that of other samples. This difference can be attributed to the following factor: molten salt offers a liquid-phase environment for grains to grow during high-temperature reactions and the process of ion transport can occur through the liquid phase. In addition, particles are able to move in a liquid medium, which can cause their collision and additional aggregation into larger particles. The particle sizes of the MASS samples are between those produced by the SSR and MSM methods. In the SSR method, prolonged high temperatures (typically lasting several hours) regulate the crystal growth phase, promoting an increase in grain size. However, the reaction process in the MASS method differs significantly from that in the SSR method, as it usually proceeds much faster and more intensely. This phenomenon can be attributed to two factors. Firstly, activated carbon has a relatively high microwave absorption coefficient, which generates intense thermal radiation during microwave absorption. Secondly, the efficient heat transfer from the microwave-heated activated carbon material to the reactant allows the reactant to heat up very rapidly and potentially reach higher temperatures than those achieved by other methods. As the temperature of the reactant increases, its dielectric constant changes, allowing it to absorb microwave energy more effectively, thereby promoting a rapid reaction. Therefore, the high temperature generated by the intense exothermic reaction of the activated carbon, combined with the rapid reaction process facilitated by microwave absorption, may result in larger grain sizes in the samples [31,32]. In contrast to the solid-phase synthesis method, the sample synthesized by HM shows a relatively uniform morphology. As illustrated in Figure 3d and Figure S1d, the average grain size is approximately 9 nm. The difference in morphology and size caused by different synthesis methods further affects the luminescence properties of samples (as discussed below). In Figure S2, the elemental composition of the MSM samples was analyzed to evaluate the influence of Na^+^ and Cl^−^. It is evident that the majority of Cl^−^ ions were removed through the cleaning process; however, it is noteworthy that Na^+^ ions were detected, which likely indicates the incorporation of Na^+^ into the crystal lattice. When Na^+^ enters the lattice, it may occupy the position of the Zn^2+^-Zn^2+^ ion pair due to the formation of a Na^+^-Cr^3+^ ion pair, thereby introducing additional traps. To further examine the elemental distribution, the EDS mappings are shown in Figure S3. All the elements (Zn, Ga, O, Cr) exhibit a uniform distribution among microparticles prepared by MASS. Additionally, since activated carbon was used as the reaction medium in the MASS method, the distribution of the C element was further analyzed using EDS. Figure S4 shows that, in addition to the C from the conductive glue on the sample platform, there is a very minor distribution of carbon on the particle surface. This may not only be attributed to the limited EDS resolution of equipment, but also the possible pollutants of activated carbon that subsequently reached the reagent. The presence of a small amount of carbon may not affect its luminous properties seriously.

**Figure 4:**
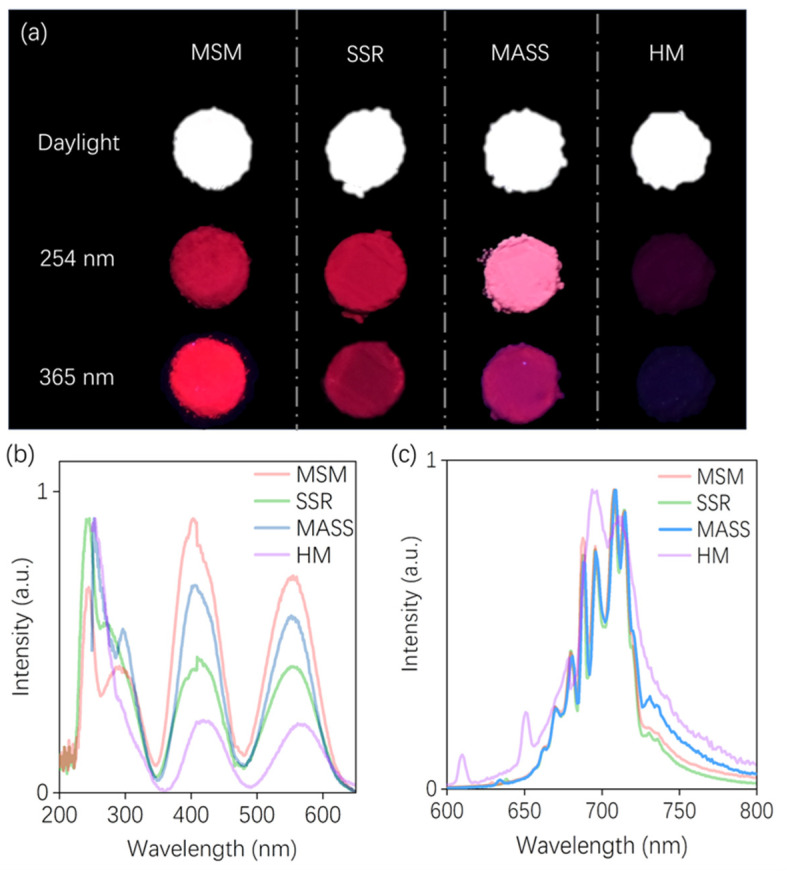
(**a**) Photographs of ZGO: Cr^3+^ upon daylight, 254, and 365 nm excitation, prepared by MSM, SSR, MASS, and HM. Photoluminescence excitation (PLE) spectra monitored at 709 nm (**b**), and photoluminescence (PL) spectra excited at 410 nm (**c**).

To directly show the luminescence intensity of samples prepared by different methods, the photographs of ZGO: Cr^3+^ upon daylight, 254, and 365 nm excitation are shown in Figure 4a. Samples prepared through MSM, SSR, MASS, and HM emitted deep-red light when exposed to 254 and 365 nm commercial LED irradiation. It is worth noting that the luminescence intensity of the sample prepared by HM is the weakest among these four synthesis methods. To further investigate the effects of different methods on the luminescence properties of ZGO: Cr^3+^, the PLE spectra were measured by monitoring the emission intensity at 709 nm, as displayed in Figure 4b. There are three absorption bands in the PLE spectra, peaking at about 250 nm, 420 nm, and 550 nm, which could correspond to the spin-allowed transitions ^4^A_2_→^4^T_1 (4P)_, ^4^A_2g_→^4^T_1g (4F)_, and ^4^A_2g_→^4^T_2g_, respectively. The PL spectra upon excitation with 410 nm blue light are shown in Figure 4c. All samples exhibit a broad emission band with several sharp lines in the NIR area (650–800 nm). The sharp line at approximately 690 nm could be attributed to the well-known ^2^E_2g_→^4^A_2g_ spin-forbidden transition, and the broadband with several peaks could probably be attributed to spin-allowed ^4^T_2g_→^4^A_2g_ of Cr^3+^ ions [33,34]. Similar to the previous results, the sample synthesized by the HM exhibits inferior luminescence properties (with a lower signal-to-noise ratio) due to their excessively small grain sizes compared with other samples synthesized by MSM, SSR, and MASS. To further investigate the impact of grain size, the SSR sample was ball-milled for 0, 1, 4, 10, and 17 h; their corresponding PLE and PL spectra are illustrated in Figure S5. The sample without ball milling exhibits a higher luminescence intensity, while the luminescence intensity gradually decreases with increasing ball milling time. This observation further emphasizes that the reduction in grain size adversely affects luminescence performance. In addition, the DRS of samples prepared by the four synthesis methods are given and the band gaps (E_g_) are calculated to be approximately 4.28, 4.63, 4.47, and 4.27 eV, as shown in Figure S6.

**Figure 5:**
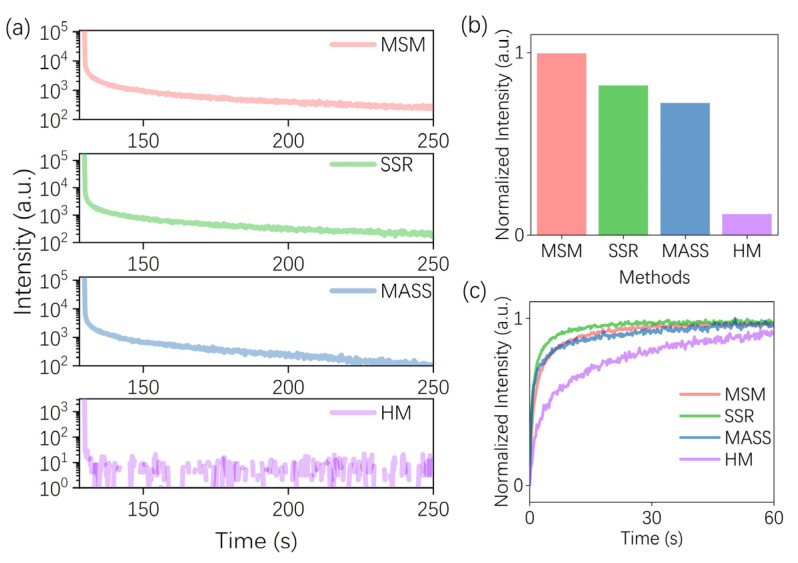
(**a**) PersL decay curves of ZGO: Cr^3+^ (λ _ex_ = 254 nm and λ _em_ = 709 nm); (**b**) comparison of the total PersL intensity of different synthesis methods; (**c**) the charging behavior of samples by MSM, SSR, MASS, and HM.

ZGO: Cr^3+^ phosphor possesses excellent PersL as a NIR PersL material, and the PersL decay in samples prepared through various methods revealed distinct PersL properties. In Figure 5a, a series of PersL decay profiles at 709 nm were observed after 2 min of charging at 254 nm LED, indicating well-preserved PersL across all samples. To further explore the influence of synthesis methods on PersL properties, a comparison of PersL intensity is presented in Figure 5b. The PersL intensity of MSM is about 1.2, 1.4, and 5 times that of SSR, MASS, and HM, respectively. The results show that the MSM sample presents a longer PersL time than the others. Simultaneously, the impact of different synthesis methods on the PersL charging process is investigated in Figure 5c. The results show a gradual increase in PersL intensity during the charging process until equilibrium is reached, indicating that most traps are filled and an equilibrium state of electron trapping and releasing is achieved. In this process, the charging speed of the SSR sample is the largest. The MSM and MASS samples have a similar charging process. The charging speed of the HM sample is clearly slower than that of the SSR sample. The differences in the PersL time and the charging process indicate an obvious influence on trap distribution in phosphors by the varied synthesis processes.

**Figure 6:**
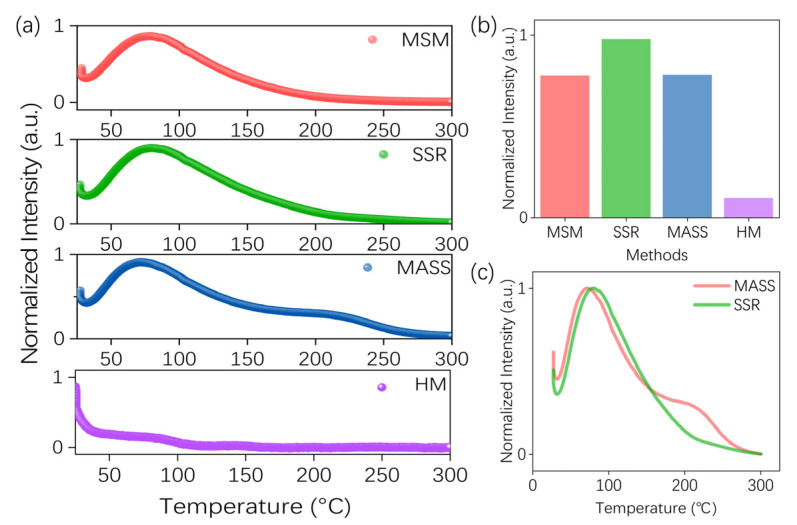
(**a**) TL glow curves (excited by 254 nm LED, monitored at 350–900 nm, the heating rate was kept at *β* of 1 °C s^−1^) of ZGO: Cr^3+^ prepared by MSM, SSR, MASS, and HM. (**b**) Comparison of the total TL intensities of different synthesis methods. (**c**) Comparison of TL glow curves of MASS and SSR samples.

To further reveal the influence of synthesis processes on PersL performance, TL glow curves of samples prepared by different synthesis methods were analyzed, as shown in Figure 6. Figure 6a demonstrates that the TL glow curves of samples synthesized via MSM, SSR, MASS, and HM exhibit similarities in the shallow trap region. The trap depth was calculated using the following formula [35]:

*E_trap_* (eV) = (−0.94 ln*β* + 30.09) *kT_m_* (2)

where the symbol *T_m_* represents the temperature at which the TL glow curve reaches its maximum, *β* represents the experimental heating rate, and k is the Boltzmann constant with a value of 8.617 × 10^−5^ eV/K. The trap depth of samples was calculated at about 0.919 eV. The total TL intensities for different synthesis methods are also depicted in Figure 6b. The total trap intensity of HM is approximately five times lower than that of SSR, which is attributed to the smaller grain size of samples as discussed above. When the grain size is too small, on the one hand, HM leads to the introduction of OH groups into the volume of NPs, which are effective luminescence quenchers together with surface acceptors for small NPs [29,36]; on the other hand, the number of traps that can effectively store energy is reduced. To further verify it, the SSR sample was subjected to ball milling for a variety of durations. Charging and TL tests were conducted on the milled samples, as shown in Figures S7 and S8. It is clear that the total charging intensity and TL intensity decrease along with increasing milling time and decreasing grain size.

The total trap intensity of SSR samples is higher than those of MSM, MASS, and HM samples; however, SSR exhibits a shorter PersL time compared to MSM. Figure S9 shows a detailed comparison of TL glow curves for MSM and SSR samples. Although the SSR sample has a larger number of total traps, more suitable and shallower traps are found in the MSM sample (see insert in Figure S9). This may be due to the fact that MSM can achieve better ion mobility in the molten solution and achieve grain growth with the aid of the liquid phase environment, which makes it prone to producing shallow traps. It indicates that the PersL performance is not only dependent on trap intensity but is also closely relevant to their trapping level distributions. As Figure S10 shows, the electrons are excited and move to the conduction band, and part of the electrons are captured by the shallow traps when they move back to the valence band. After being affected by the ambient temperature, the electrons stored in the shallow energy trap are released with the PersL. Therefore, MSM with an enhanced storage capability of shallow traps holds significant potential for improving the PersL properties.

As illustrated in Figure 6c, a detailed comparison of TL glow curves between SSR and MASS samples indicates that the MASS sample exhibits an additional deep trap at approximately 1.282 eV. Different from the reaction processes in MSM and SSR, the MASS method utilizes microwaves to provide the reaction driving force, resulting in a rapid and intense reaction. The rapid reaction process allows quick nucleation and growth of crystals, facilitating the formation of additional traps. A wide variety of traps created by the MASS preparation procedure may offer a novel perspective for the future development of PersL materials [37].

**Figure 7:**
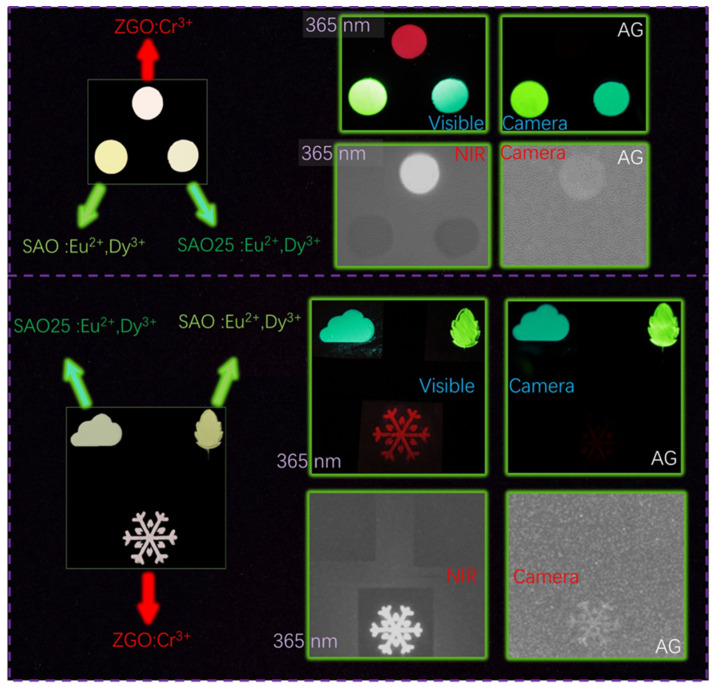
Comparison of PL (excited by 365 nm LED) and AG (pre-irradiated by a 365 nm LED for 30 s) images of SAO25:Eu^2+^, Dy^3+^, SAO: Eu^2+^, Dy^3+^, and ZGO: Cr^3+^ taken by visible and NIR cameras.

Taking advantage of the invisibility of NIR PersL to the naked eye and the good chemical stability of inorganic materials, ZGO: Cr^3+^ phosphors have great potential in night vision and information encryption applications. Figure 7 shows the PL and PersL images of ZGO: Cr^3+^ prepared via SSR compared with the SAO: Eu^2+^, Dy^3+^, and SAO25: Eu^2+^, Dy^3+^ phosphors prepared by MASS. The wafer, Leaf, Cloud, and Snowflake patterns were made by SAO: Eu^2+^, Dy^3+^, SAO25: Eu^2+^, Dy^3+^, and ZGO: Cr^3+^ phosphors. Photographs of these patterns were taken by a visible camera under daylight. Also, the pattern under 365 nm LED exhibits bright red, green, and cyan emissions (taken by visible camera). After pre-irradiation by a 365 nm LED for 30 s, the PersL images were also taken by a visible camera. The SAO: Eu^2+^, Dy^3+^, and SAO25: Eu^2+^, Dy^3+^ exhibited long-lasting green and cyan emissions, while the ZGO: Cr^3+^ seemed to show very little red light. In stark contrast, the NIR PersL can be observed via a NIR camera, which clearly shows the ZGO: Cr^3+^ patterns (in white) under a 365 nm LED. However, the patterns made by SAO: Eu^2+^, Dy^3+^, and SAO25: Eu^2+^, Dy^3+^ were not observed (shown in black). In AG (PersL) mode, the PersL image of ZGO: Cr^3+^ patterns taken by the NIR camera is clearly seen, while the SAO: Eu^2+^, Dy^3+^, and SAO25: Eu^2+^, Dy^3+^ patterns are not observed. From the above demonstrations, the ZGO: Cr^3+^ prepared by SSR exhibits great potential in information encryption and night-vision surveillance.

Based on the differences in optical properties of ZGO: Cr^3+^ obtained by different synthesis methods, the different synthesis processes could be extended to different application fields. In the field of night vision, materials are required to demonstrate reliable long- PersL, robust durability, and low-cost mass production. SSR is a synthesis process characterized by simplicity and low cost, and it effectively produces micron-sized particles with excellent crystallinity and stable physical and chemical properties. The PersL materials prepared using SSR usually have good PL and PersL properties. Consequently, the long-PersL materials obtained through SSR are suitable for night lighting applications. MSM, as an advanced preparation process, is utilized to synthesize sulfides and other materials with significant environmental impacts. This MSM method has shown its advantages under strict reaction conditions (i.e., reducing gas protection) and preparing materials with more shallow defects compared to the SSR sample. Despite the increased complexity of the synthesis process compared to SSR, MSM exhibits substantial potential for application in night lighting due to its enhanced material properties. For materials with a large number of traps, the position and distribution of traps typically determine the diversity of luminescence modes to achieve multi-dimensional information encryption. The above results indicate that samples prepared by MASS have more shallow traps and generate additional deep traps. These results indicate that the luminescent materials synthesized via MASS can possess multi-dimensional information encryption capabilities, which is highly desirable for applications in this domain. For in vivo imaging purposes, stringent size requirements of particles are needed. Intravenous injection for in vivo imaging requires small particle sizes to prevent issues such as blood vessel blockage, while micron-scale particles generally result in poor biocompatibility. The HM method can produce nano-scale long-PersL materials, which effectively meet the needs of biological imaging and medical diagnosis fields. To develop materials with enhanced properties, the HM microwave method was considered as an alternative to the traditional hydrothermal method, which utilizes microwave processing of the precursor in a closed container. This approach can effectively enhance the luminescence performance of the material and produce a stable aqueous colloidal solution, thereby meeting the requirements of biological applications [38,39]. In future studies, further attempts may be made to combine different methods, such as integrating MSM with MASS by utilizing NaCl fluxes in a MASS reactor, leveraging the liquid phase environment of the molten salt method and the rapid reaction rate of the microwave method in an effort to improve the luminous properties of the material. The integration of different synthesis processes is significant for the advancement of future material systems.

## 4. Conclusions

In summary, four groups of ZGO: Cr^3+^ persistent phosphors were prepared using MSM, SSR, MASS, and HM approaches. Investigations of the micro/nano-structure, luminescence, and PersL characteristics reveal that the properties of NIR PersL performance are largely modulated by different synthesis methods. MSM enhances PersL energy storage by 1.2 times compared to SSR due to the addition of shallow traps. HM can produce nano-scaled PersL materials with particle sizes smaller than 10 nm. However, excessively small sizes significantly reduce the total amount of traps, which is only one-fifth of the trap quantity found in SSR samples. In the future, combining this method with others could yield improved synthesis processes. MASS shows its intense and rapid reaction process to effectively generate deep traps. Taking ZGO: Cr^3+^ as an example, the study of different effects of synthesis methods on PersL performance provides a good guideline for the rapid and reasonable selection of synthesis preparation methods in the future and further promotes the development of diverse applications of materials.

## Data Availability

The data supporting the findings of this study are available from the corresponding author upon reasonable request.

## Supplementary Materials

The following supporting information can be downloaded at: https://www.mdpi.com/article/10.3390/nano14191613/s1, Figure S1: The FESEM and TEM images of ZnGa_2_O_4_:Cr^3+^ phosphors prepared by (a) MSM, (b) SSR, (c) MASS, and (d) HM; Figure S2: The EDS elemental analysis of phosphor prepared by MSM; Figure S3: The FESEM mapping of ZnGa_2_O_4_:Cr^3+^ phosphors prepared by MASS; Figure S4: The FESEM mapping of C in ZnGa_2_O_4_:Cr^3+^ phosphors prepared by MASS; Figure S5: The PLE spectra (a) and PL spectra (b) of ZnGa_2_O_4_:Cr^3+^ samples after ball milling for different times; Figure S6: The band gap (Eg) of ZnGa_2_O_4_:Cr^3+^ prepared by (a) MSM, (b) SSR, (c) MASS, and (d) HM; Figure S7: The charging behavior of SSR samples after ball milling for 0, 1, 4, 10, or 17 h; Figure S8: (a) The TL glow curves of SSR samples after ball milling for 0, 1, 4, 10, and 17 h. (b) Comparison of trap intensity within these samples from (a); Figure S9: Comparison of the TL glow curves of samples prepared by MSM and SSR methods (Insert gives an enlarged view of the selected area); Figure S10: Proposed mechanism of the trapping and de-trapping processes of phosphors.
