# Supplementary material for: Modulating Near-Infrared Persistent Luminescence via Diverse Preparation Approaches
Source: Nanomaterials (Basel). 2024 Oct 9;14(19):1613. doi: 10.3390/nano14191613 (PMC11478689; doi:10.3390/nano14191613)
Supplement: Supplementary file 1 [file nanomaterials-14-01613-s001.zip › nanomaterials-3215521-supplementary.pdf]

nanomaterials

Supporting Information

Modulating Near-Infrared Persistent Luminescence via Diverse Preparation Approaches

Xiaomeng Wang, Hengli Zhu, Yan Liu, Jingyuan Li, Lejia Cao, Jiaren Du * and Hengwei Lin *

International Joint Research Center for Photo-Responsive Molecules and Materials, School of Chemical and Material Engineering, Jiangnan University, 214122 Wuxi, China; 7220611009@stu.jiangnan.edu.cn (X.W.); 6230609013@stu.jiangnan.edu.cn (H.Z.); 1052220206@stu.jiangnan.edu.cn (Y.L.); 1052220130@stu.jiangnan.edu.cn (J.L.); 1052210127@stu.jiangnan.edu.cn (L.C.)

* Correspondence: jiaren.du@jiangnan.edu.cn (J.D.); linhengwei@jiangnan.edu.cn (H.L.)

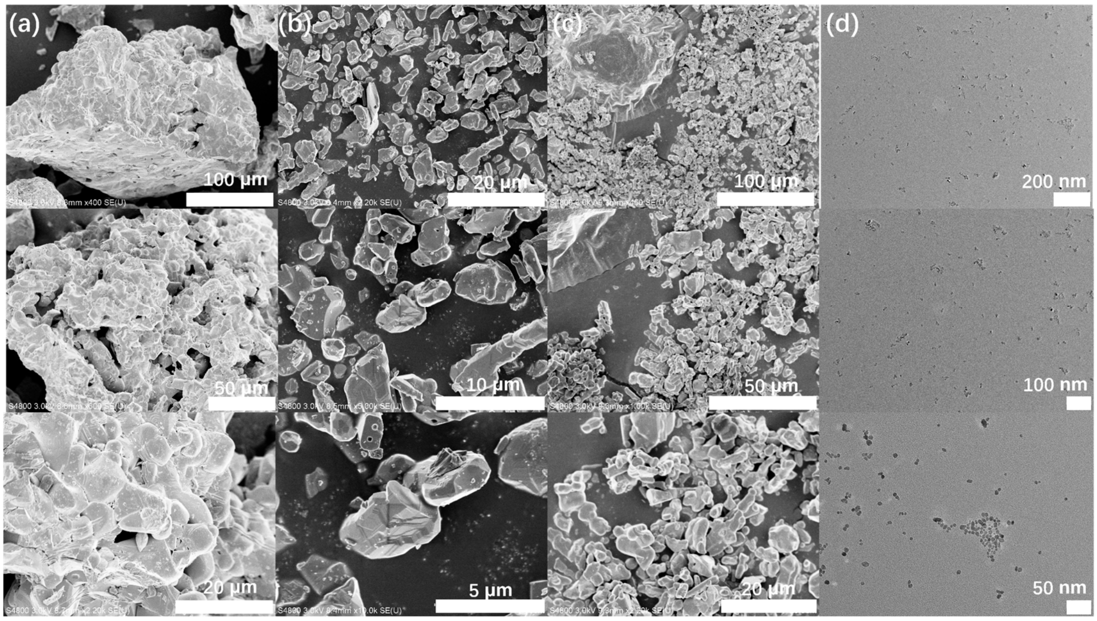

Figure S1. The FESEM and TEM images of $ZnGa_2O_4{:}Cr^{3+}$ phosphors prepared by **(a)** MSM, **(b)** SSR, **(c)** MASS, and **(d)** HM, respectively.

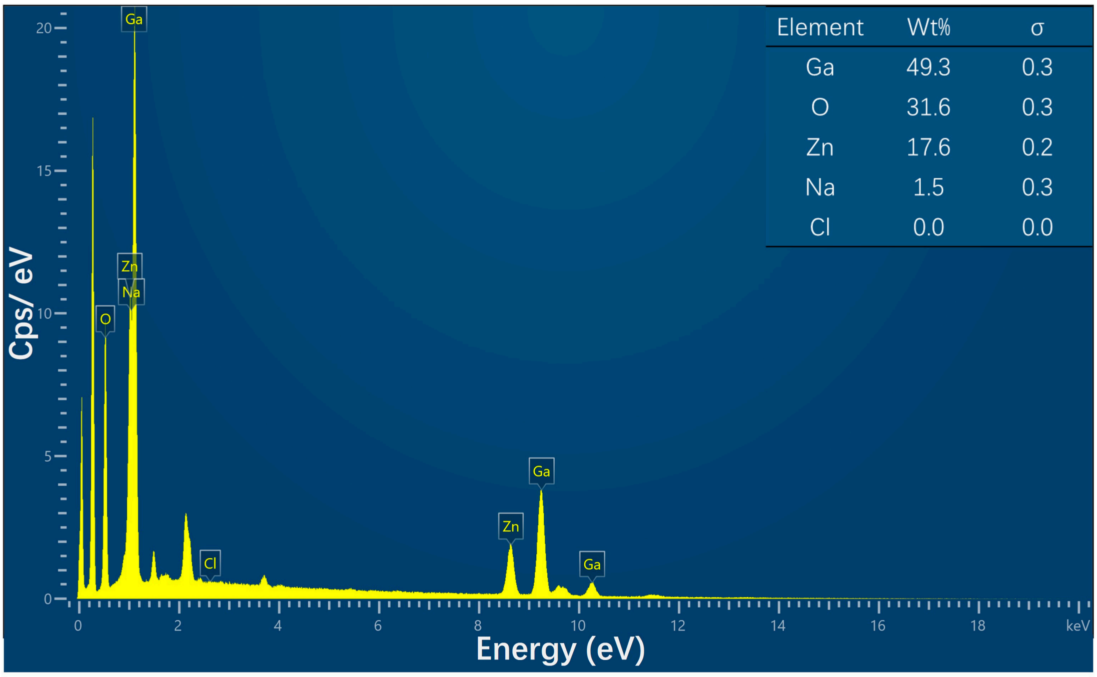

Figure S2. The EDS elemental analysis of $ZnGa_2O_4:Cr^{3+}$ phosphor prepared by MSM.

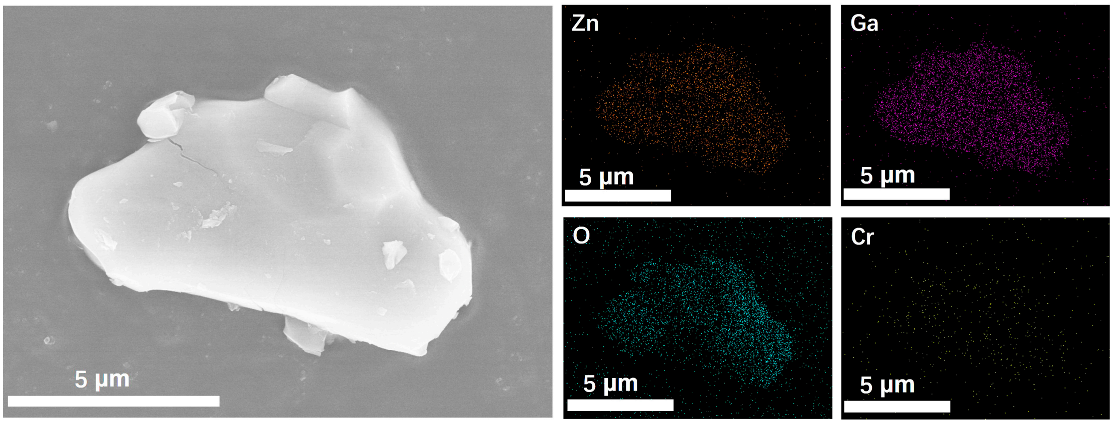

Figure S3. The FESEM image and EDS mappings of $ZnGa_2O_4:Cr^{3+}$ phosphors prepared by MASS.

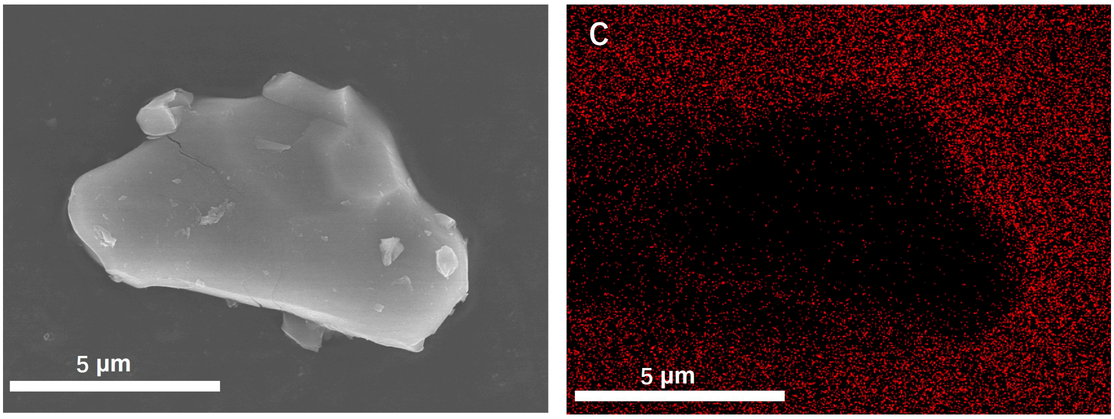

Figure S4. The FESEM mapping of element C in $ZnGa_2O_4:Cr^{3+}$ phosphors prepared by MASS.

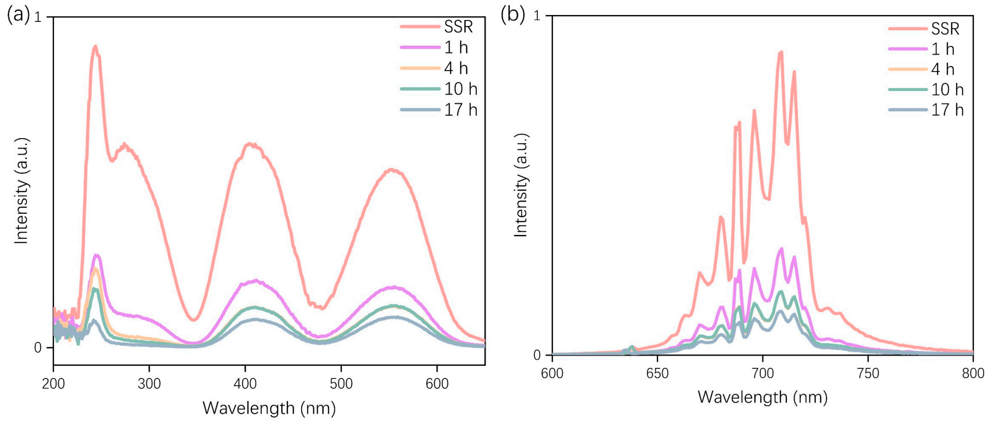

Figure S5. The PLE spectra **(a)** and PL spectra **(b)** of $ZnGa_2O_4:Cr^{3+}$ samples after ball milling for different times.

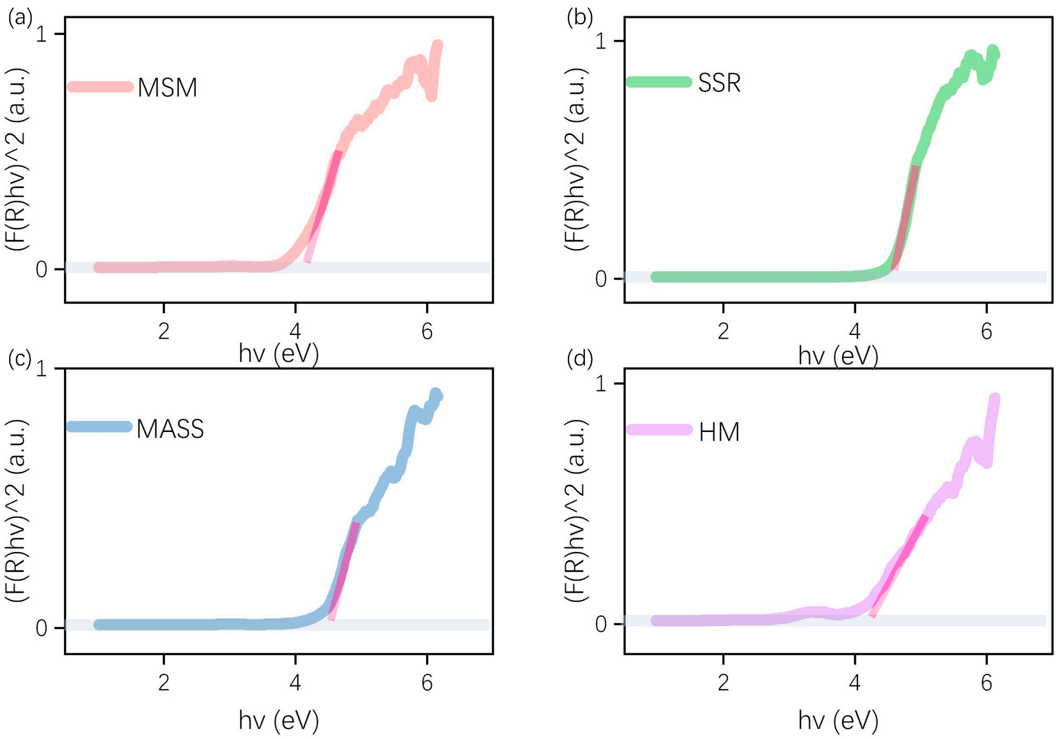

Figure S6. The bandgap (E_g) of $ZnGa_2O_4:Cr^{3+}$ prepared by **(a)** MSM, **(b)** SSR, **(c)** MASS and **(d)** HM.

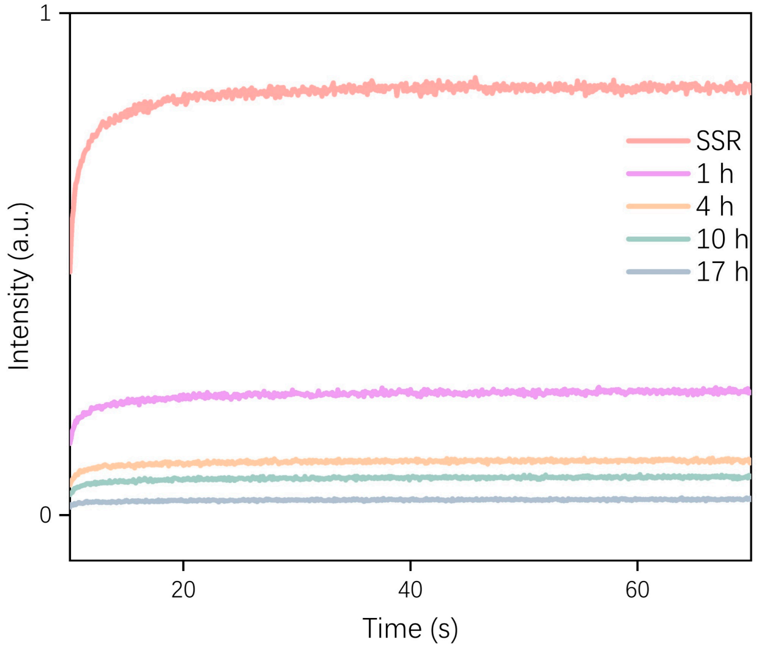

Figure S7. The charging behavior of SSR samples after ball milling for 0, 1, 4, 10, or 17 hours.

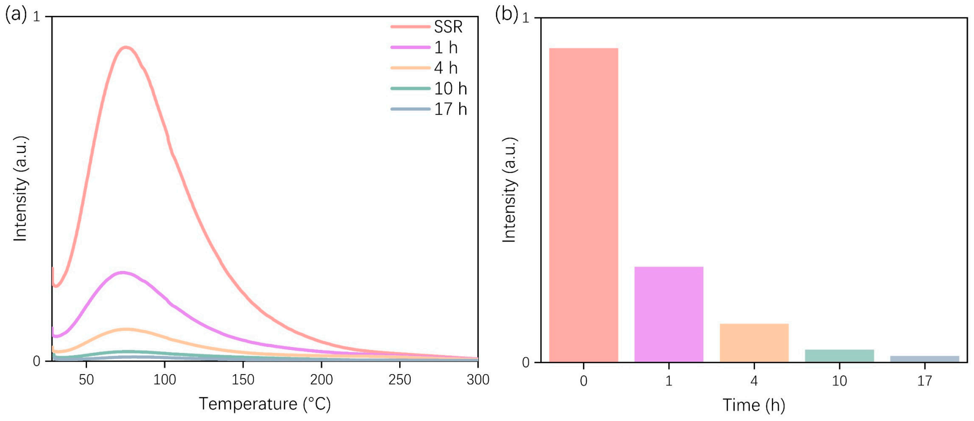

Figure S8. (a) The TL glow curves of SSR samples after ball milling for 0, 1, 4, 10, and 17 h. **(b)** Comparison of trap intensity within these samples from (a).

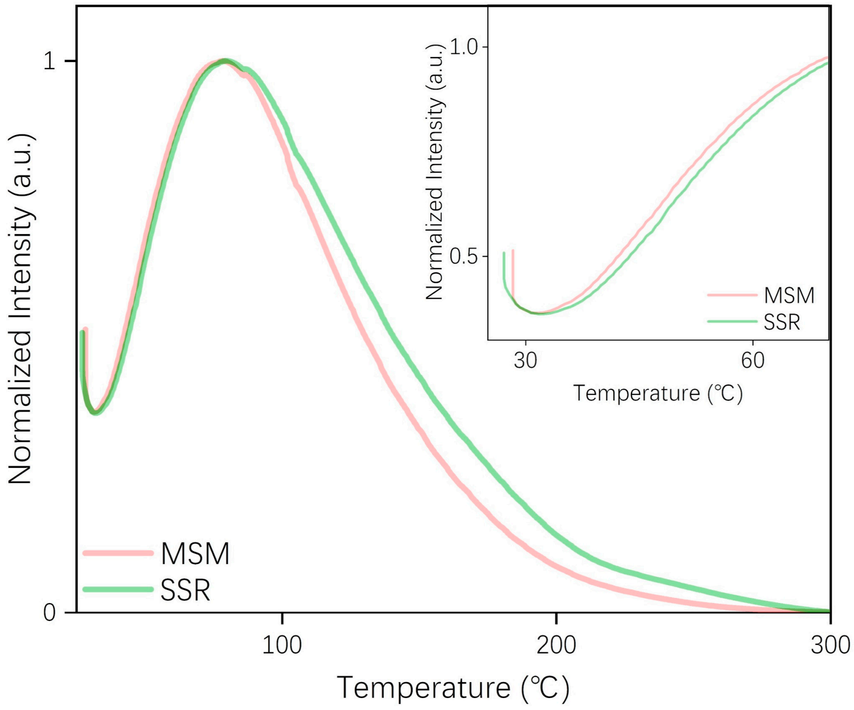

Figure S9. Comparison of the TL glow curves of samples prepared by MSM and SSR method (Insert gives an enlarged view of the selected area).

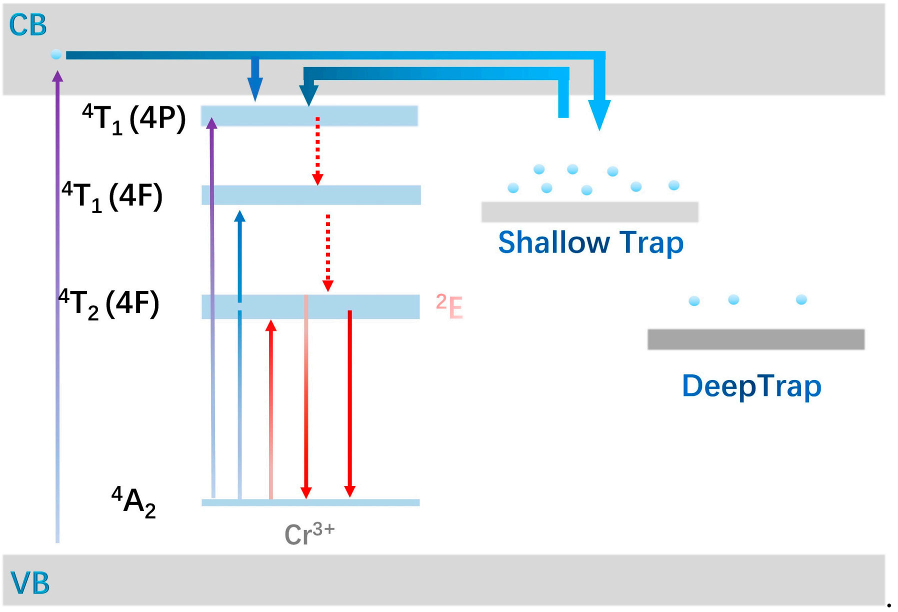

Figure S10. Proposed mechanism of the trapping and de-trapping processes in phosphors.
